# Measuring Fitness of Kenyan Children with Polyparasitic Infections Using the 20-Meter Shuttle Run Test as a Morbidity Metric

**DOI:** 10.1371/journal.pntd.0001213

**Published:** 2011-07-05

**Authors:** Amaya L. Bustinduy, Charles L. Thomas, Justin J. Fiutem, Isabel M. Parraga, Peter L. Mungai, Eric M. Muchiri, Francis Mutuku, Uriel Kitron, Charles H. King

**Affiliations:** 1 Center for Global Health and Diseases, Case Western Reserve University, Cleveland, Ohio, United States of America; 2 Department of Pediatrics, Case Western Reserve University, Cleveland, Ohio, United States of America; 3 Center for Health Care Research and Policy, Case Western Reserve University, Cleveland, Ohio, United States of America; 4 Department of Nutrition, Case Western Reserve University, Cleveland, Ohio, United States of America; 5 Division of Vector-Borne and Neglected Diseases (DVBND), Ministry of Public Health and Sanitation, Nairobi, Kenya; 6 Department of Environmental Studies, Emory University, Atlanta, Georgia, United States of America; Brown University, United States of America

## Abstract

**Background:**

To date, there has been no standardized approach to the assessment of aerobic fitness among children who harbor parasites. In quantifying the disability associated with individual or multiple chronic infections, accurate measures of physical fitness are important metrics. This is because exercise intolerance, as seen with anemia and many other chronic disorders, reflects the body's inability to maintain adequate oxygen supply (*VO_2_* ma*x)* to the motor tissues, which is frequently linked to reduced quality-of-life in terms of physical and job performance. The objective of our study was to examine the associations between polyparasitism, anemia, and reduced fitness in a high risk Kenyan population using novel implementation of the 20-meter shuttle run test (20mSRT), a well-standardized, low-technology physical fitness test.

**Methodology/Principal Findings:**

Four villages in coastal Kenya were surveyed during 2009–2010. Children 5–18 years were tested for infection with *Schistosoma haematobium (Sh)*, malaria, filaria, and geohelminth infections by standard methods. After anthropometric and hemoglobin testing, fitness was assessed with the 20 mSRT. The 20 mSRT proved easy to perform, requiring only minimal staff training. Parasitology revealed high prevalence of single and multiple parasitic infections in all villages, with *Sh* being the most common (25–62%). Anemia prevalence was 45–58%. Using multiply-adjusted linear modeling that accounted for household clustering, decreased aerobic capacity was significantly associated with anemia, stunting, and wasting, with some gender differences.

**Conclusions/Significance:**

The 20 mSRT, which has excellent correlation with *VO_2_*, is a highly feasible fitness test for low-resource settings. Our results indicate impaired fitness is common in areas endemic for parasites, where, at least in part, low fitness scores are likely to result from anemia and stunting associated with chronic infection. The 20 mSRT should be used as a common metric to quantify physical fitness and compare sub-clinical disability across many different disorders and community settings.

## Author Summary

Reduced physical fitness, which is a manifestation of the body's inability to maintain adequate oxygen supply to the tissues, can have many causes. In developing countries, a person's low physical fitness is often the result of anemia and undernutrition, which have multifactorial etiologies including poor diet and chronic infections such as malaria, hookworm and schistosomiasis. In past surveys, exercise tolerance has been measured using non-standardized tests that were poorly-suited to young children. In this study, we implemented a well-validated and reliable 20-meter shuttle run test in a low-resource area of Kenya. Results for 1950 children, aged 5–18 years, showed that impaired fitness was common and associated with anemia and poor growth with boys being more affected than girls. The 20 mSRT is a feasible and low-cost tool that can be easily delivered in low-resource settings to identify children who manifest the disabling but often sub-clinical manifestations of their disease. We propose its implementation as a standard fitness test in less developed areas to allow comparisons across morbidity studies assessing the impact of different interventions.

## Introduction

In the context of chronic disease, exercise intolerance due to decreased physical fitness is a measurable outcome strongly related to decreased quality of life in many spheres of human performance. Among children, loss of physical fitness is associated with anemia, chronic inflammatory conditions, and inadequate nutrition leading to impaired growth [1]–[3]. Accurate, affordable measurement of physical fitness is expected to become a very valuable tool in gauging community burden of disease where such conditions are common. However, to date, no fitness-related standardized test has been widely adopted as a morbidity assessment tool.

Exercise intolerance due to anemia and chronic parasitic diseases such as schistosomiasis can lead to chronic disability in children and to decreased adult productivity. These important morbidities have been routinely underestimated in past disease burden assessments [4], [5].Quantifying physical fitness can be done by many different methods, but their implementation and frequency of use most often relate to the resources available at the site of testing. In developed countries, measuring school-age children's fitness is a common practice, and well-controlled studies have been carried out to measure the maximum aerobic capacity and to compare pre- and post-training fitness results [6]–[8]. By contrast, in low resource settings, adequate assessment of aerobic capacity has rarely been done outside the research laboratory environment, and even then, a variety of different methods have been applied, with sometimes conflicting results. Reduced aerobic capacity was documented in Ethiopian and Indian children using cycle ergometry [9], [10]. Other studies have utilized portable accelerometers [11] or the Harvard-Step test [12]–[15] as fitness measuring tools. But these were neither designed nor standardized for children under 17 years old. In Mozambique, a combination of observation and activity questionnaires [16] showed no correlation with morbidity outcomes such as undernutrition. Given the variability of results for the methods employed to date, there is an evident need for a rapid, affordable physical fitness test that can be uniformly implemented in many settings around the world.

**Figure 1:**
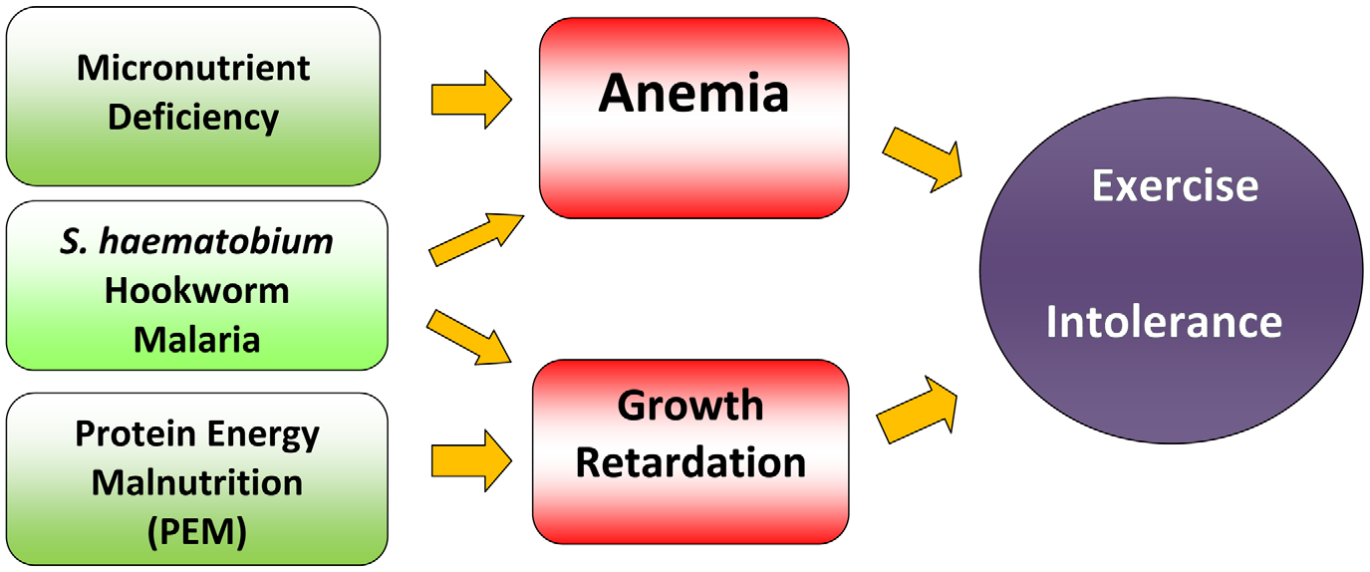
Proposed causal pathway of anemia and growth retardation manifesting as exercise intolerance due to polyparasitic diseases in conjunction with malnutrition.

In this paper we present the results from cross-sectional surveys in four parasite-endemic villages in coastal Kenya, during which participating children were uniformly tested using the well validated, low-technology multistage 20-meter shuttle run test (20 mSRT). Our working hypothesis, as shown in Figure 1, was that children affected by infection-related anemia or undernutrition would be exercise intolerant (with decreased aerobic capacity), and that this could be reliably quantified by the 20 mSRT. To the best of our knowledge this is the first time that the 20 mSRT has been used in a low-resource environment in parasitic disease research.

## Methods

### Ethics statement and eligibility criteria

Ethical clearance was obtained by the Institutional Review Board at the University Hospital Case Medical Center of Cleveland and the Ethical Review Committee of the Kenya Medical Research Institute (KEMRI). Children were eligible if they were residents of the area for at least two years, were between 5–18 years old, and they had provided child assent and written parental consent.

### Study area and population

**Figure 2:**
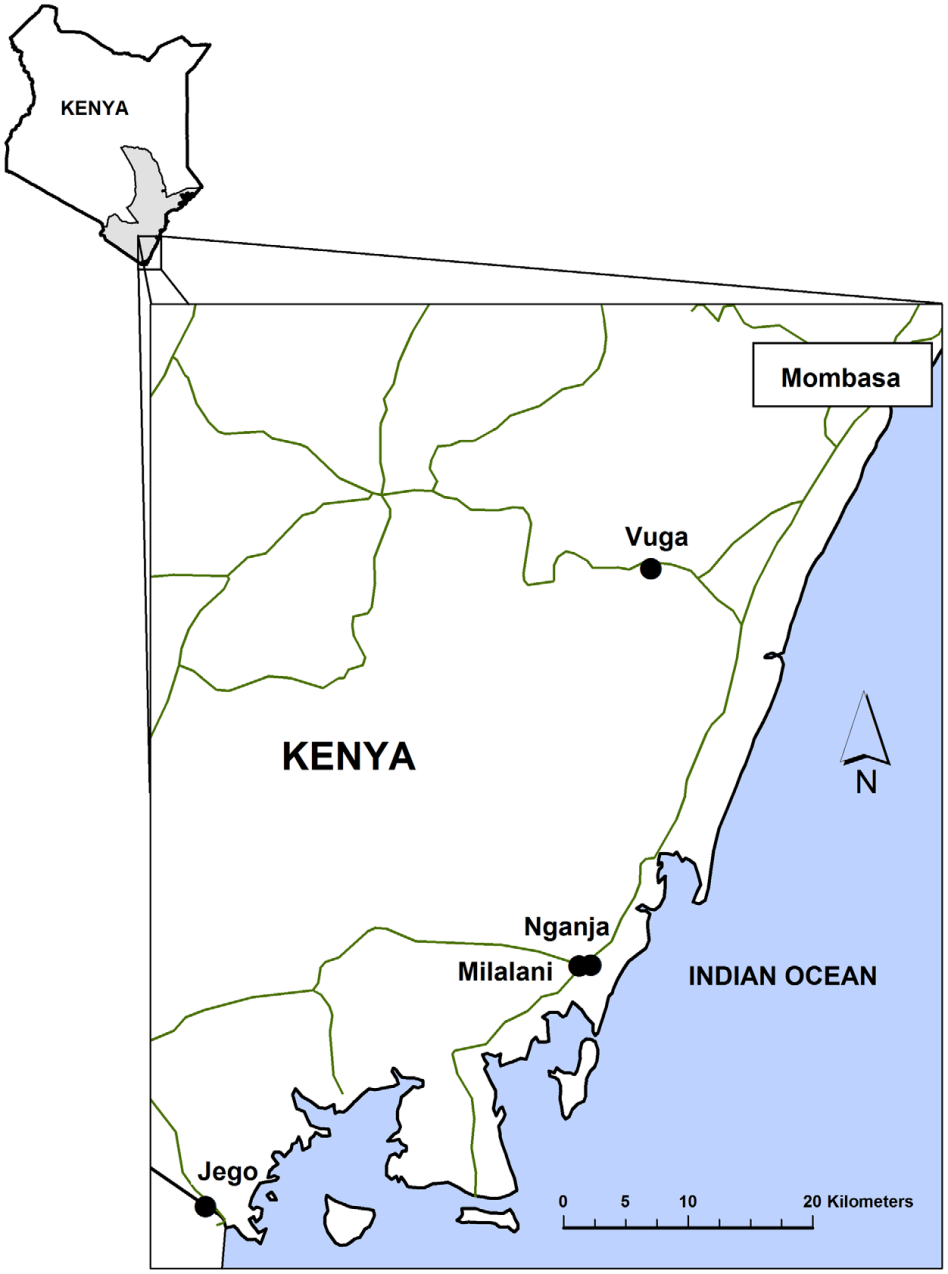
Map of the study area with the villages surveyed.

This cross-sectional study was conducted in four *Schistosoma haematobium* (*Sh*) endemic rural villages (Nganja, Milalani, Vuga, and Jego, see Figure 2) in Msambweni and Kwale Districts in Coast Province, Kenya [17]. This sub-study is part of a larger project in the area studying the ecology of transmission of vector borne parasitic infections. 78% of all eligible children in Nganja (N = 240/309), 51% (N = 416/822) in Milalani, 74% in Vuga (N = 726/983) and 73% in Jego (N = 652/890) agreed to participate. Non-participating children either i) belonged to households that refused to take part in the study or ii) did not complete all the phases involved. A common reason for refusal during the recruitment period was fear of being tested for Human Immunodeficiency Virus (although this was not part of the study) or refusal to handle the biological samples necessary for parasitology testing.

Subjects were enrolled at the time of the village demography survey in February, August and November of 2009 for Nganja, Milalani and Vuga respectively and March of 2010 for Jego. After an initial interview with the head of the household, in which general information about living conditions was obtained, children were screened for the presence of *Sh*, *Wuchereria bancrofti*, malaria and geohelminths (*Ascaris lumbricoides, Trichuris trichiura,* and hookworms). Nutritional and fitness assessment were also done. When available, dates of birth were cross-checked with national identification cards for adults and vaccination cards for children.

### Urine examination

Egg burden for *Sh* was assessed by the urine filtration method [18], and the presence of hematuria was also recorded. The subjects provided a single sample of a mid-morning urine specimen that was immediately processed.

### Stool examination

The night prior to the parasitology survey, eligible subjects were given a stool container by local community health workers to provide a single stool sample. The following morning, the stool samples were taken to a central facility and examined in duplicate by Kato-Katz method for microscopic detection of parasite eggs.

### Blood collection and processing

Finger prick blood was used to obtain a hemoglobin measurement (Hemocue, Ångelholm, Sweden), and a rapid antigen test for *P. falciparum* for malaria (ICT Diagnostics, Australia) and *W. bancrofti* for filarial (Binax, Portland, ME). Testing was performed in all participating children except one. *Anemia* was categorized according to WHO criteria by age and sex [19].

### Standardization procedures for anthropometric assessments

Because growth is considered the best indicator of nutritional status in children, standardized measurements of height and weight were taken. Prior to working in the surveys, all technical staff who performed anthropometric measurements received standardization training followed by independent reliability assessment. Supervised by a trained anthropometrist, candidates performed duplicate measurements of height (agreement within 0.5 cm), weight (agreement within 1.0 kg), for ten healthy volunteer children on the same day. The results were then compared for inter and intra-observer reliability. The trainees' intra- and inter-examiner technical errors of measurement fell within the reference values [20]–[22] and were therefore considered accurate.

### Anthropometric measurements

Eligible children were measured according to procedures described by Jeliffe [23] while wearing a kanga- a traditional light cloth- wrapped around their bodies. Weight was obtained by digital weight-scale (SECA model 803, Hanover, MD) and was rounded to the nearest 0.1 kg. Height was measured with the use of a stadiometer (SECA model 214, Hanover, MD) and measurements were read to the nearest 1.0 cm. Instruments were calibrated daily prior to use. Every measurement was performed twice and the mean values were used for analysis. Reference population Z-scores were calculated for each subject's height-for-age (HAZ) and body-mass index for age (BAZ) using two different reference populations for comparison: First, the standards included in the US Centers for Disease Control and Prevention's Epi Info™, Version 3.5.1 (CDC, Atlanta, GA) from the year 2000 [24] and second, the World Health Organization's Anthro-Plus for ages 5-19 years old (WHO, Geneva, Switzerland) with reference growth standards from the year 2006 [25], [26].

HAZ is considered an indicator of long-term linear growth whereas BAZ variations better reflect acute changes in nutritional status. According to WHO standards [27], *stunting* was categorized as an observed HAZ that was two or more standard deviations (SD) below average (HAZ score≤−2). Children were categorized as clinically *wasted* if their BAZ was more than two SD below average for their age (BAZ score≤−2). Children were further identified as *severely wasted* if their BAZ was≤−3.

### Exercise testing

**Figure 3:**
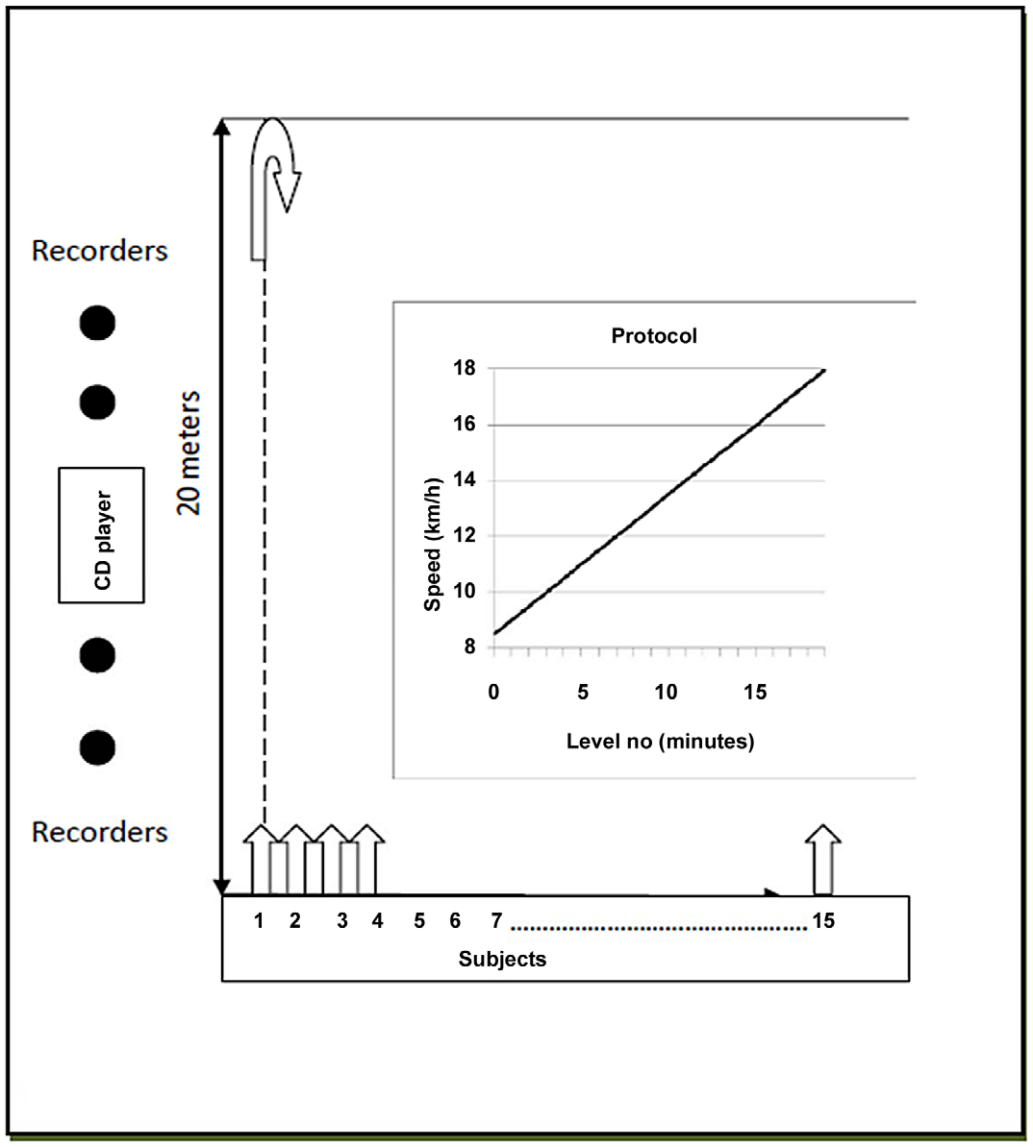
Field protocol set up for the 20 m shuttle run test adapted from Leger et. al (29).

The 20 meter shuttle run test (20 mSRT), initially validated in Canadian schoolchildren, was used to determine the maximal aerobic capacity of children enrolled in this study [28], [29]. The test was carefully explained to the participating children before the start. A demonstration was then performed by one of the recorders. Materials needed for the test included: a measuring tape to mark the 20 meter track, a battery-powered CD player, study-ID coded score sheets, and pencils. A table or desk was usually provided by the village school to facilitate data recording. The schematic diagram of the test is illustrated in Figure 3. Briefly, a group of 5–15 children were instructed to run back and forth on a 20 meter course that had been previously marked off in a flat, shady area of the village. A soccer field was used in 2 of the 4 villages. Although there was often a mixture of ages and body sizes, this did not interfere with the performance of the test, as every individual was able to run up to his or her personal limit. The number of children simultaneously performing the test was determined based on the number of recording staff available. After several pilot runs, it was concluded that every observer could effectively monitor 4–5 children per test. The subjects each lined up behind the starting line and waited for the starting beep on the pre-recorded CD. They then had to run towards the marked 20 m line, reaching it before (or at the same time as) the second beep sound signal from the CD. The interval of these signals progressively shortened, increasing the pace by 0.5 km h^−1^ every minute, from a starting speed of 8.5 km h^−1^. Each change of level, and thus speed, was announced every minute by the pre-recorded CD. Each cohort of children took between 5–11 minutes to complete the test. When the subject could no longer follow the pace, he/she was asked to stop, and their highest level obtained was recorded as the fitness score in a Study ID coded sheet. Higher scores correlated with better fitness, and these were later translated into standardized estimates of maximum oxygen consumption (*VO_2_*max) [28] for each child.

### Data management and statistical analysis

Demographic data collected in the field were double entered in hand-held devices (Dell Axim, Round Rock, Texas) using Visual CE 10 (Cambridge, MA) and a paper form. Data were then transferred in duplicate into ACCESS 2007 (Microsoft, Seattle, WA) and the databases compared for errors. Parasitology and anthropometric data were similarly entered to complete the database.

Exploratory analysis started with univariate distributions followed by bivariate analyses to explore the pairwise relationships of individual outcomes (Table S1). Egg counts were log-converted to adjust for their skewed distribution. Later multivariable analysis was used to assess associations controlling for age, gender, infection, co-infection status and intensity and a scale of socioeconomic status derived using Principal Component Analysis (PCA) of combined asset scores [30]. Linear regression modeling of fitness scores was performed to obtain estimates of their relationship to other subject variables measured in the study. Generalized Estimating Equation (GEE) modeling was used to account for household clustering effects. Multiple regression diagnostic analyses were performed (R^2^, C(p), MSE) to aid in the choice of the optimal model. Variance Inflation Factor (VIF) was also calculated to test for co-linearity among model variables.

The model outcome of interest was fitness level (as scored on the 20 m SRT) and the final GEE-linear regression models presented here were fit to establish its multiply-adjusted association with age, anemia, wasting, and stunting (using WHO age-and sex-based definitions). Additional alternative variables that were explored during model development included continuous variables for weight, height, other observed anthropometrics, hemoglobin, and/or hookworm and schistosomiasis burden (using log-transformed egg counts).We also explored the use of dichotomous/polyotomous variables for malaria or filaria infection, and infection intensity categories (light/moderate/heavy) for hookworm and schistosomiasis. All analyses were performed using SAS 9.2 (Cary, NC) and SPSS 17 (Chicago, IL).

## Results

### Participation

Of the 2034 children 5–18 years old who participated in the surveys, 1950 children (95.9%) with complete parasitological data completed the 20 m SRT, and were included in the final analysis. Seven children refused to run, and 23 (1.1%) were unable to participate due to limiting physical conditions that included asthma, seizures, pregnancy, club-foot, leg wounds and feeling unwell. Thirty-six (1.8%) did not wait for fitness testing and left after providing their biological samples.

### Performance

After a brief explanation and demonstration of the test, study participants were batched in groups of 5–15 individuals for testing. The running surface was dirt and most of the children ran barefoot, with which they typically felt most comfortable. There was a very good overall understanding of the test, with occasional need for repetition of instructions and a few false starts. When this happened, a rest period was instituted that lasted between 15–20 minutes. In performing the test, several situations were taken into consideration: i) If a child was lagging behind the recorded marked pace he/she was asked to stop and the level obtained was recorded accordingly, ii) If a false start happened, all children were asked to re-start, iii) If tripping occurred, the child was asked to stop and after a recovery period he/she restarted the test.

### Study group demography and parasitology

**Figure 4:**
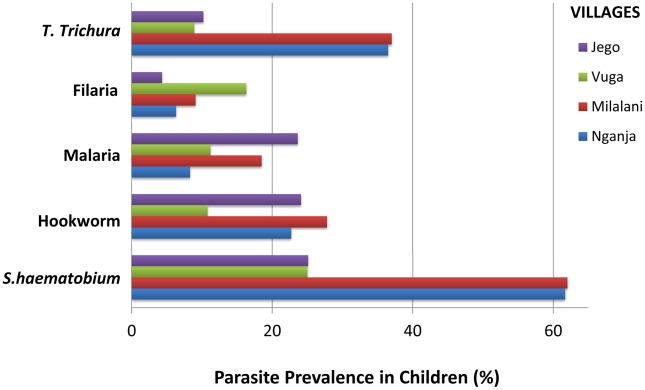
Parasite prevalence in each of the four villages surveyed.

No significant differences were observed among the four villages in terms of sex or age distribution, however significant inter-village differences were observed in the prevalence of anemia, mean hemoglobin and malnutrition parameters as shown in Table 1 **.** Village-specific parasite prevalence is illustrated in Figure 4. Prevalence of *Sh* was highest (25%–62%) followed by geohelminths (15–35%), *P. falciparum* (9–24%) and *W. bancrofti* (4–9%).

**Table 1:** Distribution of demographic, hematologic, and anthropometric features of 2034 children in four study villages in Msambweni and Kwale districts, Coast Province, Kenya.

VILLAGES(Number studied)	TOTAL(N = 2034)	NGANJA(N = 240)	MILALANI(N = 416)	VUGA(N = 726)	JEGO(N = 652)	*P* * *value*
**DEMOGRAPHIC**						
MEAN AGE (RANGE)	11.0 yr (0.2–19)	11.2 yr (5–19.5)	11.1 yr (5–19)	11.6 yr (2–19)	10.4 yr (0.2–18.2)	0.0715
% FEMALE	48%	44%	51%	51%	46%	0.0852
**HEMATOLOGIC**						
% ANEMIC+	51%	48%	51%	45%	58%	0.0567
MEAN HEMOGLOBIN (RANGE)	11.7 g/dL (3.4–18.9)	11.7 g/dL (4.8–17)	11.6 g/dL (6.3–15.4)	11.8 g/dL (5.2–16.4)	11.9 g/dL (3.4–18.9)	0.0217
**ANTHROPOMETRIC**						
% WASTED#	19%	19%	13%	31%	10%	<0.001
% STUNTED ^	36%	45%	35%	43%	25%	<0.001
% SEVERELY WASTED**	6.4%	3.3%	2.9%	13.5%	2%	<0.001

*P value refers to significance of differences among the villages by ANOVA or chi-square testing. Statistically significant differences indicated in **bold.**

**+:** Anemia based on WHO age-specific hemoglobin (Hb) criteria (19): for ages<12 yr, Hb<11.5 g/dL; for ages≥12 yr, Hb<12 g/dL; but for males≥15 yr, Hb<13 g/dL.

# Wasting: < = −2 in BMI-for-age Z score (BAZ).

^Stunting: < = −2 in height-for-age Z score (HAZ).

**Severely Wasted: < = −3 in BAZ, based on WHO 2006 growth standards (25).

### Anthropometrics and nutritional status

Both acute and chronic undernutrition were present in each of the four communities, as measured by wasting and stunting prevalence, respectively (see Table 1). Growth references from the CDC-2000 [24] as well as from the newer WHO-2006 [25] were used for comparison. When both results were compared, statistically significant differences in nutritional scoring were detected between these two standard references, with CDC growth standards indicating higher levels of undernutrition when compared to WHO growth standards (data not shown).

### Association between 20 m SRT exercise results and individual infection and nutritional status

As was expected, based on growth physiology, there were marked gender differences in 20 m SRT performance scores, so the final analysis of fitness outcomes is stratified here by sex. (Mean scores per age group for boys and girls are shown in Table S2 in the supporting information files). Because inter-village variations in fitness outcomes were small, all village data were merged and included in a single morbidity model. A separate model was run to compare estimated *VO_2_* max results for Kenyan children with the reference Canadian cohort [29]. Initial bivariate parameter estimates for each potential explanatory variable, along with their 95% confidence interval (accounting for household clustering) are shown in Table 2. Several multivariable modeling strategies were then explored, which revealed co linearity between infection and anemia consistent with the pathways to exercise intolerance shown in Figure 1. For this reason, infection status was ultimately excluded from the final model. The results from the best-fit Generalized Estimated Equation (GEE) multivariable modeling are summarized in Table 3.

**Table 2:** Associations between 20 m SRT exercise scores (VO_2_ max) and age, anemia, nutritional status, or infection status–Bivariate analyses stratified by sex.

	*Bivariate Association*	
*Parameter*	*Estimate*	*95% CI*
**Age in years**		
*Males*	**0.257**	**(0.21, 0.30)**
*Females*	**0.080**	**(0.04, 0.11)**
**Anemia** a		
*Males*	**−0.671**	**(−0.98,−0.35)**
*Females*	**−0.299**	(**−0.53, −0.05)**
**Stunting** b		
*Males*	**−0.731**	**(−1.06, −0.39)**
*Females*	−0.253	(−0.51, 0.01)
**Wasting** c		
*Males*	**−0.382**	**(−0.75, −0.01)**
*Females*	−0.199	(−0.51, 0.11)
**S. haematobium Intensity** d		
*Males*	0.026	(−0.08, 0.13)
*Females*	0.083	(−0.01, 0.17)
**Hookworm Intensity** e		
*Males*	−0.073	(−0.22, 0.07)
*Females*	**−0.124**	**(−0.24, 0.00)**
**Malaria Infection** f		
*Males*	0.194	(−0.22, 0.60)
*Females*	0.130	(−0.21, 0.47)
**Filaria Infection** f		
*Males*	−0.006	(−0.50, 0.49)
*Females*	−0.114	(−0.47, 0.24)

a Anemia based on WHO age-specific hemoglobin (Hb) criteria (19): for ages<12 yr, Hb<11.5 g/dL; for ages≥12 yr, Hb<12 g/dL; but for males≥15 yr, Hb<13 g/dL.

b Wasting:< = −2 in BMI-for-age Z score (BAZ);

c Stunting: < = −2 in height-for-age Z score (HAZ); based on WHO 2006 growth standards (25).

d *S. haematobium* intensity as log transformation of individual egg count in 10 mL of urine.

e Hookworm intensity as log transformation of individual egg count in 1 gm of stool.

f Malaria/Filaria infection scored as present or absent by rapid antigen detection card.

Estimates in **bold** are significantly associated with increased or decreased exercise scores. (*P*<0.05).

**Table 3:** Multivariable GEE linear modelling with exercise performance score as outcome, stratified by sex.

*Multivariable-adjusted linear model of 20 m SRT fitness score accounting for household clustering via generalized estimating equation*		
*Parameter*	*Estimate*	*95% CI*
**Age in years**		
*Males*	**0.314**	**(0.27,0.35)**
*Females*	**0.095**	**(0.05,0.13)**
**Anemia** a		
*Males*	**−0.282**	**(−0.56, −0.04)**
*Females*	**−0.279**	**(−0.52, −0.03)**
**Stunting** b		
*Males*	**−1.007**	**(−1.31, −0.69)**
*Females*	**−0.310**	**(−0.60, −0.01)**
**Wasting** c		
*Males*	**−0.829**	**(−1.20, −0.45)**
*Females*	−0.302	(−0.67,0.07)

a Anemia based on WHO age-specific hemoglobin (Hb) criteria (19): for ages<12 yr, Hb<11.5 g/dL; for ages≥12 yr, Hb<12 g/dL; but for males≥15 yr, Hb<13 g/dL.

b Stunting: < = −2 in height-for-age Z score (HAZ); based on WHO 2006 growth standards (25).

c Wasting: < = −2 in BMI-for-age Z score (BAZ); Estimates in **bold** are significantly associated with increased or decreased exercise scores. (*P*<0.05).

For **girls**, in bivariate analysis, being anemic and harboring hookworm infection negatively correlated with fitness scores (estimates of −0.299 and −0.124 respectively, Table 2). Increasing age significantly associated with better aerobic capacity (estimate 0.080 per year of age). When adjusted for other covariates (Table 3), *stunting* reached significance (estimate −0.310) and both anemia and age remained as significant predictors of fitness score.

For **boys**, bivariate correlation analysis indicated a positive association between increasing age and better fitness scores (estimate 0.257 per year), as had been seen with girls. Malnutrition parameters and anemia negatively correlated with fitness scores, both in bivariate as well as multivariable analysis (*stunting* adjusted estimate-1.007; *wasting* adjusted estimate −0.829; *anemia* adjusted estimate −0.282). After this multivariable adjustment, no additional association was seen between fitness and parasite infection *per se*.

**Figure 5:**
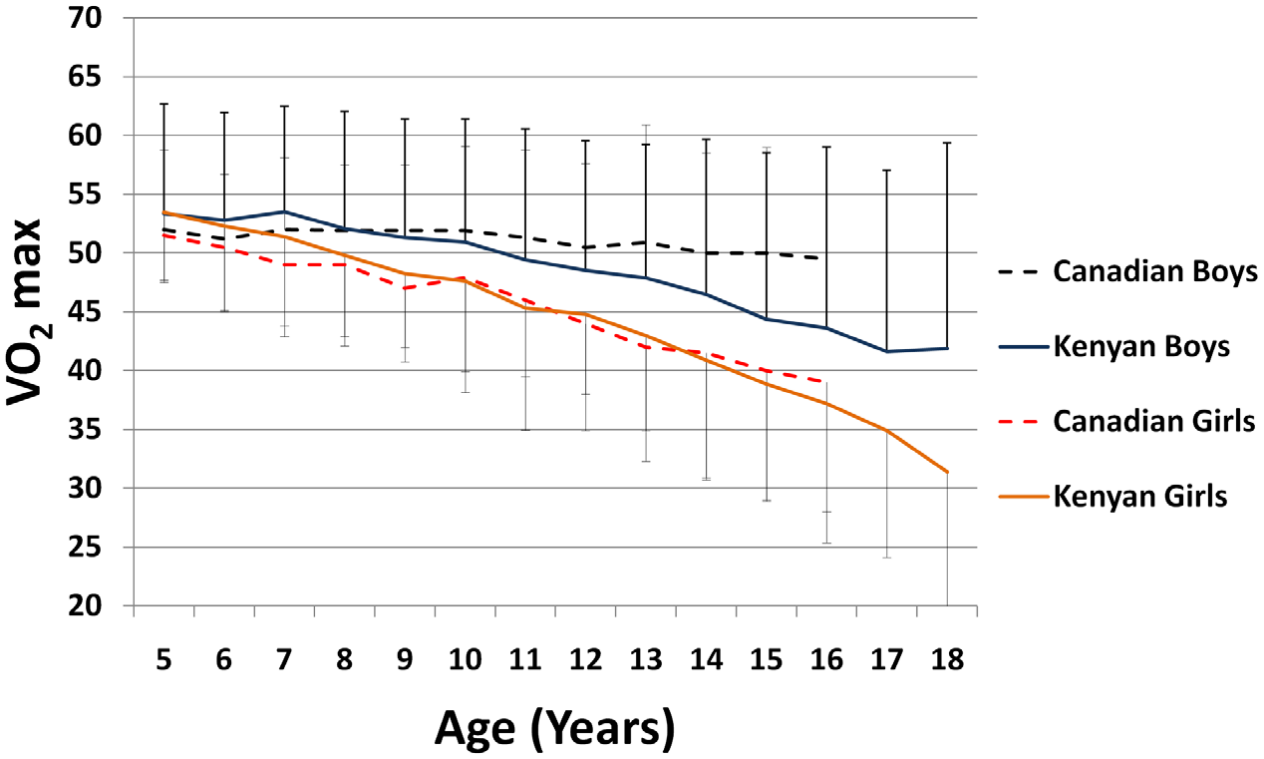
VO_2_ max differences between Canadian and Kenyan children by sex.

*VO_2_ max outcomes.* As detailed in Table S2 **,** there were marked differences in mean fitness scores according to age and sex. Boys manifested a relatively constant *VO*_2_ max (at about 51 ml kg^−1^ min^−1^) from ages 5–10 in all villages (Figure 5). This was, however, followed by an evident decline to 42 ml kg^−1^ min^−1^ by 18 years of age. The girls' results show a progressively descending *VO*_2_ max with age, from 53 ml kg^−1^ min^−1^ at 5 years to 32 ml kg^−1^ min^−1^ at 18 years old. Kenyan boys, when measured against a reference group of Canadian boys, performed comparably up to age 10, followed by a relatively sharp decline in *VO*_2_ max relative to the Canadian standard**.** Canadian and Kenyan girls had few differences across all age groups (Figure 5).

## Discussion

There is a need for a standardized aerobic fitness test in epidemiological surveys. In the past, fitness has been measured among children harboring parasitic infections in several different ways, but none have proven to be both sufficiently easy to perform and reliable so as to become a standard test for field studies [1], [11], [16], [32]. The Harvard Step Test (HST) has been one of the most widely implemented field tests in previous studies. For example, it has been used to monitor changes in fitness scores after treatment of schistosomiasis and geohelminths in Kenya [13], [33]. In Brazil, the HST has been used to document the association between diarrheal disease and decreased fitness [1]. In Zimbabwe it was used to assess physical work performance and improvement in adult cane cutters after anti-schistosomiasis treatment [14] and in China it was a useful tool in advanced adult *Schistosoma japonicum* disease [15]. However, the HST has never been formally validated in younger children. Because the HST requires accurate timing and pulse taking, the number of children who can be tested per day is limited, and there are questions about its operational limitations in terms of inter- and intra-observer variability. Our group pilot tested the HST before exploring other exercise options, and found that there was a wide inter and intra-recorder variation in the required pulse measurements at 1, 5, and 10 minutes post-exercise.

Accelerometers have also been used to measure spontaneous activity [11], but activity does not necessarily represent fitness [34]–[36]. Physical fitness relates to a normal physiological functional capacity allowing the individual to have adequate oxygen supply to the tissues. For the accelerometer approach, one must consider that cultural differences contribute to different activity patterns for boys and girls, and these differences need to be accounted for in the interpretation of activity data. Questionnaires have been used for activity assessments in Mozambique, but were found to have limited utility for estimating energy expenditure [16]. Other tests addressing strength and endurance have been used for testing adolescent girls in Senegal, and these have documented apparent fitness reductions in an undernourished group [37].

Our choice of the 20 mSRT was based on its simplicity as a field test and its validity vis a vis laboratory-based physiological testing [6]–[8], [38]. Since its development by Leger and Lambert in 1982 [38], the 20 mSRT has been widely used in the developed world as a means of estimating aerobic capacity in adults and children. Its prediction of the maximum aerobic capacity (*VO_2_* max) is calculated by age and gender-adjusted linear regression from the maximum speed obtained in the test [29]. In this benchmark Canadian study, the accuracy of the 20 m SRT was validated against standard multistage treadmill testing (correlation of SRT score to the *VO_2_* max attained was r = 0.90). Test-retest reliability coefficients were 0.89 for children and 0.95 for adults [29]. Since Leger and Lambert's initial description in 1982 [38], numerous studies (all in developed countries) have reported similar test-retest reliability with applicability for large scale fitness assessments [39], [40]. In Germany, Mechelen et al. performed a study in order to counter-validate the 20 mSRT in children against direct measurements of *VO_2_* max using a multistage maximal treadmill test in boys and girls ages 12–14 years. They concluded that the 20 mSRT is a valid tool to be used to evaluate the maximal aerobic power in children [41].

With slight variations in protocol, the 20 mSRT may be the most widely used aerobic fitness field test among children and adolescents in industrialized countries [6], [7]. It has also been used in asthmatic children to monitor cardiorespiratory changes during an aerobic training program [40]. However, to our knowledge, the 20 mSRT has never been used as a disability measuring tool in a low-resource setting. Aandstad and colleagues from Norway used the 20 mSRT in Tanzania to compare aerobic fitness in Tanzanian and Norwegian children. However, this study did not take into consideration infection or other morbidity status of the participants [42].

Although we were not able to compare 20 m SRT results to formal laboratory or ergometer testing, our results conform to those obtained in Canada in correlating fitness with increasing age, increasing height, and gender differences. This strongly supports the validity and applicability of the 20 m SRT as a useful tool in gauging fitness in less-developed areas. Consistent with international testing results (summarized in a meta-analysis of 109 studies from 37 countries [8]) we found that boys, overall, had better scores than girls. Comparison between our cohorts reveal Kenyan scores to be, overall, below those obtained in Canada for children 5–18 years [28], based, primarily on boys being less fit after 10 years of age.

In our field-based studies of parasitic disease burden, we approached local validation of the 20 m SRT by examining whether sex-specific and age-adjusted fitness scores were significantly modified by physical conditions such as anemia that are known to limit physical fitness in other populations. As expected, anemic girls and boys scored significantly lower on the 20 mSRT than their non-anemic local counterparts, reflecting a measurable relative disability. Girls with hookworm infection had more exercise intolerance than boys, however this effect was not seen in the multivariable-adjusted model suggesting that the hookworm effect was mediated through anemia or nutritional variables in the model. Malaria or filarial infection were not significantly associated with aerobic capacity in either gender, but because these two infections were relatively rare, we might not have had sufficient power in the study to see an independent effect for each infection. The bivariate association seen in boys between schistosomiasis and increased fitness may reflect a selective increase in exposure to infected water in more active children, as previously described among South African boys [34]. We hope to further clarify this effect as we evaluate personal activity patterns in our expanded study.

Malnutrition parameters, in particular, stunting and wasting, emerged as strong predictors of decreased fitness, predominantly in boys. This is consistent with findings of previous studies about stunting [35], which indicate that children develop better biomechanical motor efficiency as their height increases. However, wasted boys were also significantly less fit, underscoring the importance of assessing both types of acute and chronic measures of nutritional status when measuring disability.

There were limitations to our study. First, this cross-sectional study can only indicate significant associations with reduced fitness, but cannot indicate causality. We did not approach the comprehensive measurement of fitness testing in terms of aerobic capacity, strength, flexibility, or body composition. However, we were able to establish that the 20 m SRT is feasible, and appears to be reliable in a rural, resource-limited setting, with minimal requirements for observer and participant training. As a limitation of our analysis, misclassification of infection prevalence by underdiagnosis of active infections may have limited our ability to establish a direct association between infection status and fitness. Given that our diagnoses were based on screening parasitology of a single day's blood, urine or stool specimens using methods known to have incomplete sensitivity [18], [43]–[46], parasite prevalence (as well as infection intensity) was, no doubt, underestimated in our study. In our future analysis, we plan to refine our estimates of past and present infection with specific anti-parasite antibody detection [47].

In summary, this study was able to link fitness, as measured by a low-technology field test, with prevalent anemia and growth stunting, which are known morbidity outcomes affecting children with chronic parasitic infections. We believe the results presented in this paper can serve as a point of reference for other projects aiming to measure fitness in low-resource settings. Prior international standardization of the 20 mSRT makes it an especially valuable tool for refining estimates of disease burden in less-developed countries. Beyond its use in epidemiological research, it could be easily implemented in rural schools as a screening fitness test to detect underlying health conditions, as is commonly done in industrialized countries. Through simplified detection of sub-clinical morbidity at the community level, children at risk for anemia and/or associated parasite infections could then be brought to earlier medical attention, thereby enhancing the impact of national control programs.

## Supporting Information

Table S1 Pearson correlation between exercise level obtained and covariates of interest.
(DOCX)

Table S2 Exercise level, resulting speed, and VO_2_ max. Summary means and standard deviations by age and gender.
(DOC)
