# Supplementary material for: Measuring Fitness of Kenyan Children with Polyparasitic Infections Using the 20-Meter Shuttle Run Test as a Morbidity Metric
Source: PLoS Negl Trop Dis. 2011 Jul 5;5(7):e1213. doi: 10.1371/journal.pntd.0001213 (PMC3130006; doi:10.1371/journal.pntd.0001213)
Supplement: Table S1 — Pearson correlation between exercise level obtained and covariates of interest. (DOCX) [file pntd.0001213.s001.docx]

**Supplemental table 1**. Pearson’s correlation between exercise level obtained and covariates of interest

| **Pearson Correlation Coefficients Prob > \|r\| under H0: Rho=0 Number of Observations** | | |
| --- | --- | --- |
|  | **ExerciseLevel**  **Girls** | **ExerciseLevel**  **Boys** |
| **Age in years** | 0.15202 <.0001 943 | 0.36581 <.0001 1007 |
| **Anemia present**  **[WHO criteria]** | -0.07937 0.0148 943 | -0.13422 <.0001 1007 |
| ***S.haematobium***  **Heavy intensity** | 0.04555 0.1627 941 | -0.01323 0.6750 1006 |
| ***S.haematobium***  **Low intensity** | 0.01132 0.7287 941 | 0.06881 0.0291 1006 |
| ***S.haematobium***  **Uninfected** | -0.04623 0.1565 941 | -0.04062 0.1980 1006 |
| **Stunting**  **[HAZ < -2, WHO criteria]** | -0.06298 0.0532 943 | -0.14313 <.0001 1007 |
| **Wasting**  **[BAZ< -2, WHO criteria]** | -0.03993 0.2205 943 | -0.06247 0.0475 1007 |
| **Hemoglobin** | 0.13870 <.0001 942 | 0.25845 <.0001 1006 |
| **Weight** | 0.10524 0.0012 943 | 0.40572 <.0001 1007 |
| **Height** | 0.19417 <.0001 943 | 0.53920 <.0001 1007 |
| **Hookworm**  **Egg count** | -0.07109 0.0296 937 | -0.06979 0.0277 995 |
| ***S.haematobium***  **Egg count** | 0.05369 0.0998 941 | 0.08730 0.0056 1006 |
| **Socioeconomic Status** | 0.04973 0.1270 943 | -0.00457 0.8849 1007 |
