# Supplementary material for: Measuring Fitness of Kenyan Children with Polyparasitic Infections Using the 20-Meter Shuttle Run Test as a Morbidity Metric
Source: PLoS Negl Trop Dis. 2011 Jul 5;5(7):e1213. doi: 10.1371/journal.pntd.0001213 (PMC3130006; doi:10.1371/journal.pntd.0001213)
Supplement: Table S2 — Exercise level, resulting speed, and VO2 max. Summary means and standard deviations by age and gender. (DOC) [file pntd.0001213.s002.doc]

**Supplemental Table 3:** Exercise level, resulting speed, and VO_2_max. Summary means and standard deviations by age and gender.

| **Age (yrs):** | **5** | **6** | **7** | **8** | **9** | **10** | **11** | **12** | **13** | **14** | **15** | **16** | **17** | **18** |
| --- | --- | --- | --- | --- | --- | --- | --- | --- | --- | --- | --- | --- | --- | --- |
| **Participants** |  |  |  |  |  |  |  |  |  |  |  |  |  |  |
| Boys (N=1007) | 49 | 88 | 87 | 86 | 75 | 90 | 71 | 81 | 107 | 82 | 62 | 70 | 48 | 11 |
| Girls (N=943) | 63 | 80 | 82 | 70 | 85 | 87 | 75 | 90 | 76 | 59 | 71 | 55 | 45 | 5 |
| **Level Obtained^*^** |  |  |  |  |  |  |  |  |  |  |  |  |  |  |
| Mean for Boys | 2.77 | 3.84 | 4.98 | 4.95 | 5.33 | 6.01 | 6.03 | 6.29 | 6.67 | 6.89 | 6.76 | 6.86 | 6.52 | 7.06 |
| Mean for Girls | 2.96 | 3.72 | 3.83 | 3.93 | 4.14 | 4.4 | 4.18 | 4.73 | 4.67 | 4.67 | 4.53 | 4.42 | 4.38 | 3.87 |
| SD- Boys | 1.49 | 1.89 | 2.04 | 2.02 | 2.22 | 2.17 | 2.13 | 2.11 | 1.92 | 2.21 | 2.21 | 2.47 | 2.4 | 1.87 |
| SD*-*Girls | 1.09 | 1.60 | 1.67 | 1.56 | 1.71 | 1.57 | 1.89 | 1.54 | 1.81 | 1.81 | 1.66 | 2.09 | 1.45 | 0.17 |
| **Speed^x^** |  |  |  |  |  |  |  |  |  |  |  |  |  |  |
| Mean for Boys | 9.38 | 9.92 | 10.47 | 10.45 | 10.65 | 10.97 | 10.97 | 11.13 | 11.32 | 11.41 | 11.37 | 11.41 | 11.24 | 11.53 |
| Mean for Girls | 9.48 | 9.86 | 9.91 | 9.96 | 10.06 | 10.19 | 10.09 | 10.36 | 10.34 | 10.31 | 10.25 | 10.21 | 10.21 | 9.93 |
| SD- Boys | 0.74 | 0.94 | 1.01 | 1.01 | 1.10 | 1.08 | 1.06 | 1.05 | 0.95 | 1.10 | 1.10 | 1.23 | 1.2 | 0.93 |
| SD*-*Girls | 0.54 | 0.80 | 0.83 | 0.78 | 0.85 | 0.78 | 0.94 | 0.77 | 0.90 | 0.90 | 0.83 | 1.04 | 0.72 | 0.08 |
| ***VO_2_ max*^**^** |  |  |  |  |  |  |  |  |  |  |  |  |  |  |
| Mean for Boys | 52.35 | 52.75 | 53.52 | 51.8 | 51.07 | 51.05 | 49.52 | 48.65 | 48.12 | 47.2 | 45.37 | 44.1 | 41.65 | 41.7 |
| Mean for Girls | 52.77 | 52.54 | 51.05 | 49.55 | 48.32 | 47.23 | 45.00 | 44.67 | 42.85 | 41.23 | 39.19 | 37.21 | 35.42 | 32.18 |
| SD- Boys | 2.99 | 3.94 | 4.41 | 4.53 | 5.15 | 5.18 | 5.25 | 5.37 | 5.03 | 5.97 | 6.14 | 7.06 | 7.01 | 5.62 |
| SD*-* Girls | 2.19 | 3.35 | 3.60 | 3.50 | 3.96 | 3.76 | 4.67 | 3.93 | 4.74 | 4.89 | 4.61 | 5.97 | 4.25 | 0.53 |

^*^Level numbers are announced every minute on a pre-recorded CD. ^x^ Speed is in km h^-1 **^Predicted *VO* _2_max ( Y, ml kg ^-1^  min ^-1^ ) is obtained from maximal shuttle run speed *( X _1_*, km h ^-1^ ) and age ( *X*_2_ ) applying the regression equation obtained from 7000 reference Canadian children aged 6-18 years old (29) : Y =31.025 + 3.238*X_1_*– 3.248 *X_2_*  + 0.1536 *X_1_ X_2 ._*
